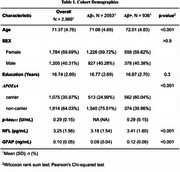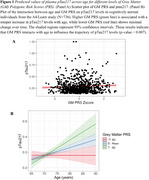# Relationship between Polygenetic Risk Score of BrainAge and plasma biomarkers in the A4/LEARN studies

**DOI:** 10.1002/alz70862_110208

**Published:** 2025-12-23

**Authors:** Jorge Garcia Condado, Colin Birkenbihl, Hannah M Klinger, Mabel Seto, Gillian T Coughlan, Michael J. Properzi, Hyun‐Sik Yang, Aaron P. Schultz, Jasmeer P. Chhatwal, Dorene M. Rentz, Asier Erramuzpe, Jesus M Cortes, Keith A. Johnson, Reisa A. Sperling, Rachel F. Buckley, Ibai Diez

**Affiliations:** ^1^ Massachusetts General Hospital, Harvard Medical School, Boston, MA USA; ^2^ Biobizkaia HRI, Barakaldo, Bizkaia Spain; ^3^ University of the Basque Country, Leioa, Bizkaia Spain; ^4^ Center for Alzheimer’s Research and Treatment, Brigham and Women’s Hospital, Harvard Medical School, Boston, MA USA; ^5^ Ikerbasque, the Basque Foundation for Science, Bilbao, Bizkaia Spain; ^6^ Melbourne School of Psychological Sciences, University of Melbourne, Melbourne, VIC Australia

## Abstract

**Background:**

The BrainAge Gap estimates the discrepancy between predicted and chronological brain age based on neuroimaging. Calculating a Polygenic Risk Score (PRS) from a BrainAge Gap estimate quantifies the genetic predisposition to accelerated brain aging. The objective of this study is to examine the association between the genetic propensity for higher or lower BrainAge Gap and plasma biomarkers of AD. Linking genetic predisposition to brain aging with early AD related changes could improve our understanding of the early disease mechanisms and risk factors

**Methods:**

We examined 3014 cognitively normal participants from the A4 and LEARN studies (71.4 ± 4.6 age; 40% male). PRS of BrainAge models were calculated for each subject using the summary GWAS statistics of Wen et. al, Nature Communications 2024 for three types of BrainAge models: Grey Matter (GM), White Matter (WM) and Functional Connectivity (FC). We focused on the following plasma biomarkers gathered at baseline: *p*‐tau_217_ (Eli‐Lilly, *N* = 736), GFAP (Roche Diagnostic, *N* = 1643) and NfL (Roche Diagnostic, *N* = 1641). We used a general linear model to study the association between each of the 3 plasma biomarkers with each of the 3 PRS measures, and additionally including the interaction between age and PRS for each BrainAge model.

**Results:**

None of the BrainAge PRS were found to be significantly different by Aβ‐PET status, *APOE*ε4 carriership, age, sex or education. BrainAge GM PRS was positively associated with *p*‐tau_217_ levels (*p* = 0.01), particularly among the older adults (*p* = 0.007). In sensitivity analyses, covaring sex, *APOE*ε4 status and years of education did not alter the results. None of the BrainAge PRS were associated with GFAP or NFL.

**Conclusion:**

Genetic factors associated with increased propensity for accelerated brain aging in the grey matter is associated with *p*‐tau_217_, an early and sensitive marker of AD. These genetic predispositions were more pronounced in older age, highlighting the importance of age as a critical factor that may interact with genetic susceptibility to brain aging, potentially through cumulative lifetime exposures, increasing vascular burden, or age‐related declines in cellular repair mechanisms. Associations solely with BrainAge GM PRS implies the specificity of accelerated brain aging in grey matter as a potential early marker of AD risk.